# P05.52 . Patient-reported experiences with adjunctive naturopathic care for type 2 diabetes in CAM-naïve patients

**DOI:** 10.1186/1472-6882-12-S1-P412

**Published:** 2012-06-12

**Authors:** E Oberg, R Bradley, K Sherman, C Hsu, C Calabrese, D Cherkin

**Affiliations:** 1 Bastyr University Research Institute, Seattle, USA; 2 Group Health Research Institute, Seattle, USA; 3 Naturopathic Physicians Research Institute, Portland, USA

## Purpose

People with type 2 diabetes (T2D) struggle with their chronic disease, and few reach recommended targets for self-care activities and clinical risk factors. We explored the experience of using adjunctive naturopathic care (ANC) in people with T2D to better understand the role ANC may play in facilitating health behavior change and improving self-care.

## Methods

A phenomenologic qualitative study was embedded within a one-year prospective observational trial of ANC for people with inadequately controlled T2D. Focus groups and key informant interviews were audio-recorded, transcribed, coded, and analyzed using an inductive content analysis approach. We explored rationale, process, and outcomes from receiving ANC.

## Results

Twenty-two of the 40 trial participants were interviewed. Participants differed non-participants only in their lower rating of satisfaction with ANC (p=0.03). The majority (64%) perceived limitations in usual care, which influenced their participation in the trial. The most striking characteristics of the experience of receiving ANC (in contrast to usual care) were the: (1) collaborative communication style, (2) holistic perspective, and (3) pragmatic approach to addressing behavioral change. Many participants reported feeling increased hope knowing they had new options and tools to take control of their health. Participants reported improved self-care behaviors, specifically, engaging in health promoting activities (diet, exercise, stress reduction), improved self-efficacy, and better understanding the impact of psychosocial factors on their self-care. These findings were corroborated with statistically significant improvements in measures of mood and behavioral change in the parent study. A minority of participants expressed uncertainty about value of dietary supplements given the out-of-pocket costs.

## Conclusion

A variety of important benefits were reported by persons with inadequately controlled T2D who tried ANC. These findings are noteworthy because the trial participants had no previous knowledge of or experience with naturopathic care.

